# Synergistic Action of Gentamicin and Bacteriophage in a Continuous Culture Population of *Staphylococcus aureus*

**DOI:** 10.1371/journal.pone.0051017

**Published:** 2012-11-30

**Authors:** Amy E. Kirby

**Affiliations:** Biology Department, Emory University, Atlanta, Georgia, United State of America; University of Birmingham, United Kingdom

## Abstract

With the increasing frequency of antibiotic resistance and the decreasing frequency of new antibiotics entering the market, interest has returned to developing bacteriophage as a therapeutic agent. Acceptance of phage therapy, however, is limited by the unknown pharmacodynamics of a replicating agent, as well as the potential for the evolution of resistant bacteria. One way to overcome some of these limitations is to incorporate phage and antibiotics into a dual therapy regimen; however, this increases the complexity of the pharmacodynamics. The aim of this study is to develop an experimental system to evaluate the pharmacodynamics of dual phage-drug therapy. A continuous culture system for *Staphylococcus aureus* is used to simulate the pharmacokinetics of periodic antibiotic dosing alone and in combination with lytic phage. A computer model representation of the system allows further evaluation of the conditions governing the observed pharmacodynamics. The results of this experimental/modeling approach suggest that dual therapy can be more efficacious than single therapies, particularly if there is an overlap in the physiological pathways targeted by the individual agents. In this case, treatment with gentamicin induces a population of cells with a strong aggregation phenotype. These aggregators also have an increased ability to form biofilm, which is a well-known, non-genetic mechanism of drug resistance. However, the aggregators are also more susceptible than the parental strain to the action of the phage. Thus, dual treatment with gentamicin and phage resulted in lower final cell densities than either treatment alone. Unlike in the phage-only treatment, phage-resistant isolates were not detected in the dual treatment.

## Introduction

Since the introduction of antibiotics in the 1940s, most research has focused on antibiotics as the optimal treatment for bacterial infections, and bacteriophage have been predominantly used as tools for molecular biology, with the notable exception of work in the former Soviet Union and Poland [1]. As the utility of antibiotics has decreased due to rising resistance, interest in developing bacteriophage as therapeutic agents has been rekindled. Recent studies have investigated the potential utility of phage therapy as treatment for a variety of human infections [2], [3], [4], [5], [6], [7], [8], [9], as a food additive [10], as a method to reduce pathogen burdens in livestock [11], [12], and as a disinfectant for industrial surfaces [13], [14], [15].

Although there is great interest in the potential for phage therapy, there are a number of concerns regarding its safety and utility [16], [17], [18], [19]. Like antibiotics, phage-resistant bacteria can evolve, and often do so quite rapidly [20], [21], [22], [23]. Although this doesn’t always eliminate the phage population, it would cause phage therapy to fail. The issue that is the most difficult to reconcile, however, is the inherent (and largely unknown) risks of introducing into patients an organism that is capable of replication and, perhaps, transmission and spread [18], [24].

To moderate the impact of some of these issues, current studies are focusing on combining phages with other therapeutics, especially antibiotics. Engineered phage have been used as gene delivery vehicles to increase bacterial susceptibility to antibiotics [25], [26], as micro-delivery vehicles to specifically target antibiotics [27], and as enzyme delivery agents to breakdown biofilms, increasing antibiotic efficacy [17], [28]. Unmodified phage can also act in concert with antibiotics by modifying the biofilm architecture to increase susceptibility [29], [30], or targeting plasmid-bearing cells, thereby reducing the frequency of plasmid-borne resistance in the population [31]. Co-administration of antibiotics could also increase the efficacy of phage therapy by stimulating increased phage production, as seen in *Salmonella enterica* and *Escherichia coli* [32], or induction of lysogenic phage, as in *S. aureus* [30].

Like multi-drug therapy, combined phage-drug therapy is less likely to fail due to resistance because bacteria resistant to one agent can still be killed by the second agent [17], [33]. Additionally, phage- and drug-resistance are unlikely to be acquired simultaneously, as can happen with multiple drug resistances carried on mobile elements [34]. Recent evidence suggests that the resistant mutants arising from combined phage-drug treatment are less virulent than resistant mutants arising from drug treatment alone [35].

In this study, the population dynamics of dual treatment with lytic bacteriophage and gentamicin were explored using *S. aureus* PS80 in a continuous culture system. To determine the critical parameters and thresholds controlling the system, a computer model that recapitulated the complex dynamics seen in the experimental system was used. The results suggest that biofilm formation is the critical intersection between the response to gentamicin and to bacteriophage. Biofilm formation allows the cells to become more refractory to killing by gentamicin. Conversely, for the bacteriophage used in this study, cells with increased biofilm-forming capacity are more susceptible to phage-induced killing. Thus, dual treatment is more effective than either single treatment, with cells driven into biofilm formation by the antibiotic only to be killed by the bacteriophage.

## Materials and Methods

### Bacteria, Bacteriophage and Antibiotics

*S. aureus* PS80, a capsulated (type 8) clinical isolate, was obtained from the American Type Culture Collection (ATCC 27700). For routine culture and density estimation, bacteria were maintained on LB agar (Fisher Scientific, Pittsburgh, PA) at 37°C.

The uncharacterized *S. aureus* bacteriophage SA5 was a kind gift from Ian Molineux. SA5 was isolated from a phage cocktail produced by the Eliava Institute of Bacteriophage, Microbiology, and Virology in Tbilisi, Georgia. The phage was amplified in host cell strain PS80 and stored at 4°C.

Gentamicin was obtained from Sigma Aldrich (St. Louis, MO), prepared as a 1% solution in sterile H_2_O, and stored at −20**°**C.

### Continuous Culture

Continuous culture systems were set up as previously described ( [20] and www.eclf.net) using ∼35 ml of cation-adjusted Mueller Hinton II (MHII) broth (Fisher Scientific, Pittsburgh, PA) as the growth medium and maintained at 37°C with vigorous aeration. Each vessel was inoculated with 100 µl of an overnight flask culture of PS80. The system was allowed to equilibrate for at least seven days to allow the cultures to reach a stable density of ∼10^9^ colony-forming units per milliliter (cfu/ml). The experiments were initiated by the addition of antibiotics, phage, or antibiotics plus phage (T = 0). The antibiotic- and dual-treated vessels were dosed with 100× MIC gentamicin every 24 hours after initiation of the experiment. One hundred microliters of phage SA5 was added to the appropriate vessels at T = 0, resulting in an initial phage density of ∼1×10^7^ plaque-forming units per milliliter (pfu/ml). At the indicated times, 0.5–1 ml samples were aseptically collected from each vessel. Viable cell densities were estimated by serial dilution in sterile 0.85% saline and plating on LB. Phage densities were estimated from the same sample dilutions by plating in soft agar lawns of PS80 on phage plates.

The experiments presented here are one replicate of five and are representative of the results observed for all of the replicates.

### Minimum Inhibitory Concentrations (MIC)

MICs were determined using a modified CLSI broth microdilution protocol [36]. Briefly, bacteria were grown overnight in MHII and inoculated into a 2-fold antibiotic dilution series in a 96-well polystyrene plate (Fisher Scientific, Pittsburgh, PA) such that the final cell density was ∼10^5^ cfu/ml. After 18 hrs incubation at 37°C with agitation, the plates were assessed by eye and by optical density. The MIC was determined to be the lowest concentration which had an OD_630 nm_ less than or equal to 25% of the antibiotic-free value or the lowest concentration with no visible clumps, whichever was higher.

For population MICs from the continuous culture vessels, samples were taken directly from the culture vessels, diluted, if necessary, to ∼1×10^5^ cfu/ml, and inoculated directly into the MIC plate containing a 2-fold antibiotic dilution series. The culture and MIC determination conditions were the same as described above.

### Determination of Phage Dynamics

Crude phage replication was assessed by inoculating a high density of cells (∼1×10^7^ cfu/ml) with a low density of phage (∼1×10^4^ pfu/ml). At 6 hrs, the viable cell densities were determined by serial dilution and plating. Phage dilutions were chloroform-treated to release mature phage particles prior to inoculation into a soft agar lawn of PS80 and plating on phage plates.

The adsorption rate of SA5 to different populations of cells was determined by tracking the loss of free phage in the presence of the cells of interest [37]. An overnight culture of bacteria was diluted to 10^7^–10^8^ cfu/ml in MHII and incubated at 37°C with shaking for 1–2 hrs to allow the cells to begin growing. At time 0, 1×10^3^ pfu/ml SA5 was added to the cells and the cultures were incubated at 37°C with shaking. At 2, 4, 6, 8, 10, 12, 14, and 20 minutes, 1 ml aliquots were removed and filtered to remove the cells and any attached phage. The filtrate was plated on a PS80 lawn to determine the remaining density of free phage. The adsorption rate (γ) was calculated from the following equation,
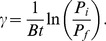
(1)where *B* is the initial bacterial density in cfu/ml, *t* is the time interval in min, and *P_i_* and *P_f_* are the initial and final phage densities, respectively, in pfu/ml [37]. This formula assumes that the bacterial density *B* is constant over the experimental time period.

One-step growth curves were used to determine the burst size and latent period of the phage [38]. The host bacteria were grown in MHII overnight. The cultures were diluted in MHII to ∼10^8^ cfu/ml. Phage SA5 was added at an MOI of ∼0.1 and allowed to adsorb for 10 min. To stop further phage adsorption, the cultures were diluted into pre-warmed MHII. The infected centers were incubated at 37°C with shaking and samples were taken at 40, 50, 60, 70 and 80 minutes. The samples were plated in soft agar lawns of PS80 on phage plates. The first time point after the plateau in phage density marks the end of the latent period. Burst size was calculated as the final phage density divided by the initial infected centers (i.e.-initial total phage – initial free phage).

### Phage Sequencing

High titer phage lysates were treated with RQ1 DNase (Promega Corporation, Madison, WI) for 30 min at 37°C to destroy contaminating bacterial DNA. Intact phage were pelleted by ultracentrifugation in an SW50.1 rotor (Beckman) at 50,000 rpm at 4°C for 45 min. Phage pellets were resuspended in 0.2 M NaCl, and the DNA was released by four consecutive extractions with an equal volume of pH-equilibrated phenol (Sigma). The DNA was precipitated with isopropanol, resuspended in sterile distilled water, and stored at 4°C.

DNA sequencing was performed by the University of Texas at Austin Institute for Cellular and Molecular Biology Genomic Sequencing and Analysis Facility using Roche 454 next-generation sequencing technology. Assembly and analysis were completed using the Lasergene 10 software package (DNASTAR, Inc., Madison, WI). The complete genome sequence is available in GenBank (Accession number JX875065).

### Computer Models

All computer simulations were programmed in the Berkeley Madonna software package and are available for download at www.eclf.net/programs.

## Results

### Population Dynamics during Single- and Dual-treatment Regimes

**Figure 1:**
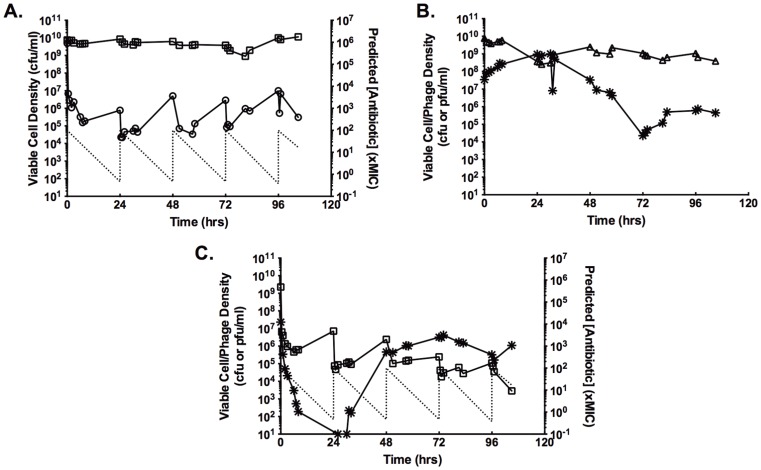
The dynamics of gentamicin, phage, and dual treatment of *S. aureus* continuous cultures. A.) Changes in cell density of a continuous culture treated with 100× MIC gentamicin every 24 hours. Squares, control; circles, GEN-treated; dashed line, predicted antibiotic concentration. B. Changes in cell and phage density in a phage-treated continuous culture. Triangles, cell density; stars, phage density. C. Changes in cell and phage density in a dual-treated continuous culture. Squares, cell density; stars, phage density; dashed line, predicted antibiotic concentration.

Continuous cultures of *S. aureus* PS80 were used as an in vitro model system for assessing the pharmacodynamics of single and dual treatment with gentamicin and bacteriophage. Without any intervention, continuous cultures of PS80 established an equilibrium density of 10^9^–10^10^ cfu/ml and this density was stable throughout the experimental period (Fig. 1A, squares). As previously reported [39], when equilibrium cultures were challenged with 100× MIC of gentamicin, there was a rapid and substantial decrease in viable cell density (Fig. 1A, circles). However, as the antibiotic concentration decreased (Fig. 1A, dashed line), the viable cell density stabilized and, eventually, began to recover, reaching a density of ∼10^7^ cfu/ml 24 hours after challenge. Repeatedly challenging the culture with 100× MIC every 24 hours established a stable oscillation in the viable cell density, with a maximum of ∼10^7^ cfu/ml and a minimum of ∼10^5^ cfu/ml.

In contrast, challenging an equilibrium density culture with a single, high-density dose of bacteriophage SA5 had very little, if any, impact on the viable cell density (Fig. 1B, triangles). Despite the high density of host cells, there was no initial burst of phage replication (Fig. 1B, stars). Instead, the phage density slowly decreased, leveling off at ∼10^6^ pfu/ml. Importantly, the rate at which the phage density waned was less than would be expected based on flow out of the system, indicating that the phage was replicating within the system.

Combining a single dose of phage with daily doses of 100× MIC gentamicin resulted in cell density oscillations very similar to those seen with the drug alone (Fig. 1C, squares). For the first 48 hrs, the oscillations were indistinguishable from those seen with drug only treatment. However, by 72 hours, the density of the dual challenged culture was detectably lower than the drug only culture, and the viable cell density continued to decrease for the remainder of the experimental period. The phage density, on the other hand, displayed dramatically different dynamics when combined with gentamicin dosing (Fig. 1C, stars). After inoculation, the phage density rapidly declined, dropping below the level of detection by 24 hours. From 24–48 hours, the phage density slowly increased. By 60 hours post inoculation, the phage had reached its equilibrium density of ∼10^6^ pfu/ml and maintained this density for the remainder of the experiment. There were no detectable differences in the plaque morphology or infection parameters of the phage isolated from late-stage dual-treated cultures and the ancestral SA5, as would be expected if dual treatment induced a resident prophage.

Previous work has shown that phage resistance arises very quickly in continuous cultures, eventually dominating the bacterial population [20]. In those conditions, the phage is maintained in a minority population of susceptible cells. For phage SA5, this does not appear to be the case. In the culture challenged with only phage, no resistant isolates were detected at 24 hrs and only 5% of isolates at 48 hrs were resistant to SA5. By 96 hrs post challenge, only 60% of isolates were phage-resistant. In the dual-treated culture, phage resistance was never detected, despite the replication and maintenance of the phage.

### Induction of an Aggregator Phenotype

**Figure 2:**
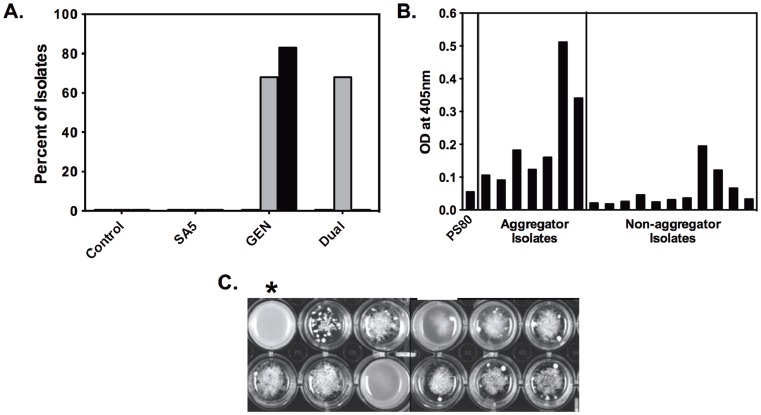
Induction of an aggregation phenotype with strong biofilm-forming capabilities. A.) Percentage of continuous culture isolates with the aggregator phenotype from each treatment category. Isolates were tested from the 0 (white), 48 (gray), and 96 (black) hrs timepoints. B.) Biofilm-forming capability of continuous culture isolates as measured by crystal violet assay. PS80 is the ancestral strain. Aggregator and non-aggregator isolates were randomly selected from isolates from the GEN- and dual-treated continuous cultures. C.) The range of aggregator phenotypes seen when isolates were grown in TSB in 24-well plates. The starred well is an example of the parental, non-aggregator phenotype.

In the continuous cultures treated with gentamicin, a new cell population was identified. Two days after the initiation of antibiotic dosing, 67% of the isolates from the gentamicin-treated cultures exhibited a strong aggregation phenotype upon subculture (Fig. 2A and C). By 96 hrs, 83% of the isolates were aggregators. Dual treatment with antibiotic and phage also induced a rise in the frequency of aggregators at 48 hrs, but, instead of continuing to increase, the new phenotype was undetectable by 96 hrs. The induction of aggregation was not in response to continuous culture or phage predation, as the control culture and the phage only culture did not produce any detectable aggregator isolates.

The aggregator isolates are a stable variant of the parental strain PS80; neither repeated subculture nor storage at −80°C changed the phenotype. The aggregator isolates were found to differ from the parental strain PS80 in their capacity to form biofilm. Using the standard crystal violet assay for biofilm formation [40], the aggregator isolates formed more biofilm than either the parental strain PS80 or non-aggregating isolates from the same culture (Fig. 2B). Despite being induced by the presence of gentamicin, the isolates capable of forming aggregates were not more resistant to gentamicin in the planktonic state. Under standard CLSI conditions for MIC determination [36], isolates with the aggregator phenotype were indistinguishable from the parental strain PS80. Indeed, “MIC creep” was not a factor under these experimental conditions; increased MICs were not found in any culture isolate nor in the collective culture populations.

### Phage Infection Parameters

To better understand the population dynamics associated with phage predation, the genome of SA5 was sequenced. The genome of SA5 contains 138,195 basepairs and is 99.89% identical to staphylococcal phage ISP, a member of the *Myoviridae,* subfamily *Spounavirinae* [41]. SA5 does not encode any of the genes associated with lysogenic phage, which is consistent with the lack of experimental evidence for lysogeny (data not shown). Indeed, ISP and other Spounaviruses are known to be strictly lytic [41].

**Figure 3:**
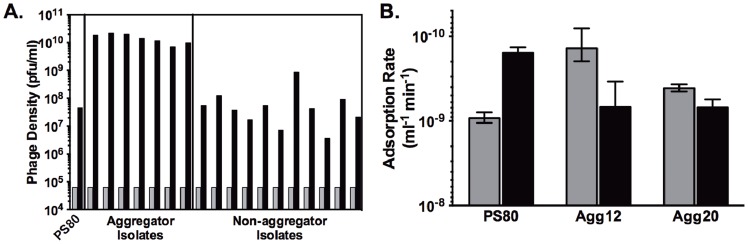
Crude phage replication rates and adsorption rates to aggregator and non-aggregator isolates. A.) Crude phage replication assay. Equivalent densities of cells were inoculated with phage SA5 (initial density, gray bars). Final phage densities (black bars) were determined after 6 hrs. B.) Adsorption rates of phage SA5 on ancestral PS80 and two independent aggregator isolates. Gray bars are the adsorption rate calculated from 2–6 mins, black bars are the adsorption rate calculated from 6–14 mins.

Phage adsorption rates were measured for the parental strain PS80 and two independent aggregator isolates: Agg12 (isolated from the drug-only culture) and Agg20 (isolated from the dual-treated culture). For all three strains, the adsorption rates were biphasic (Fig. 3B). SA5 adsorption to PS80 was initially very low (9.2×10^−10^ ml^−1^ min^−1^), and then decreased nearly ten-fold to 1.6×10^−10^ ml^−1^ min^−1^. Conversely, adsorption to Agg12 was initially more rapid at 1.4×10^−10^ ml^−1^ min^−1^, followed by an increase to 6.9×10^−10^ ml^−1^ min^−1^. While still displaying a biphasic adsorption rate, Agg20 was increased modestly, from 4.1×10^−10^ ml^−1^ min^−1^ to 6.9×10^−10^ ml^−1^ min^−1^.

Surprisingly, the burst size for SA5 in PS80 was quite low, only 4.4+/−1.5 pfu. The measured burst size increased in the aggregator strains, but not equivalently. In Agg12, the burst size increased only to 6.6+/−1.4 pfu. However, in Agg20, an isolate with a very strong aggregation phenotype, the burst size nearly tripled to 12.1+/−3.1 pfu. For all of the strains tested, the latent period was approximately 50 mins.

Crude phage replication assays were carried out to examine the combined effects of the changes in adsorption rate and burst size in the aggregator cells. Equivalent densities of exponentially growing cells were challenged with a low density of SA5 and replication was measured as the increase in phage density after 6 hrs. Compared to the parental PS80, aggregator isolates are much more efficient hosts for SA5 (Fig. 3A). On average, aggregator isolates produced over 2.5 logs more phage in 6 hours than did an equivalent density of PS80. Non-aggregating isolates from the same continuous cultures as the aggregators were indistinguishable from the parental strain.

### Modeling the Population Dynamics of the System

To better understand the dynamics of the single- and dual-treatment model systems, numerical models were developed which can be used to interpret the experimental results and develop hypotheses for the underlying mechanisms. There were three major questions that the model sought to address. 1) Can the dynamics observed with SA5 alone be explained by its infection parameters and strictly lytic life cycle? 2) Can the increased ability to form biofilm account for the rise in the aggregator population under gentamicin treatment? 3) Can the changes in phage replication parameters in aggregator strains account for the maintenance of the phage in the dual-treated cultures, where the cell density is low?

**Figure 4:**
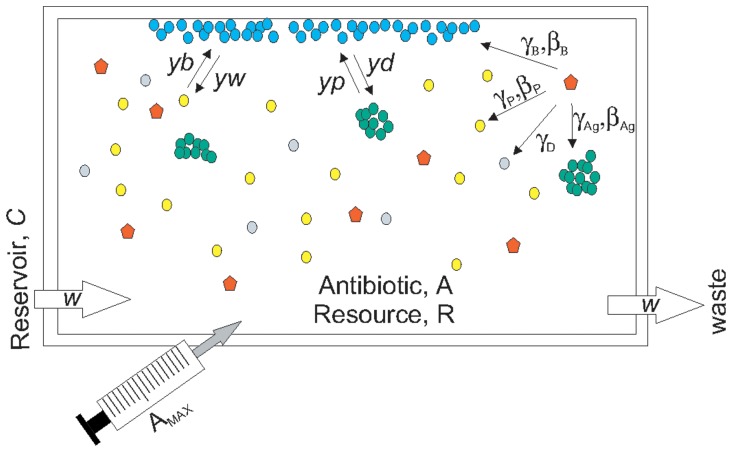
Schematic representation of numerical model of dual-treatment. Into a vessel of 1 ml volume, resource *R* flows at a rate *w* from a reservoir with a resource concentration of *C,* giving a resource concentration *R* within the vessel. Planktonic cells (*P*, yellow) can be infected by phage (*Q*, red) and are subject to flow *w*. Biofilms (*B,* blue) can be formed by switching of both planktonic and aggregator (*Ag*, green) cells, are not susceptible to flow, and can be infected by phage. Biofilm cells are lost by switching to either planktonic or aggregator cells. Aggregator cells can be infected by phage and are subject to flow. Dead cells (*D*, gray) can bind phage non-productively and are subject to flow. Phage, when present, is subject to flow. In antibiotic-treated trials, the drug is added at concentration *A_MAX_* every 24 hours. Dilution, flow out of the system and decay determine the antibiotic concentration *A* in the vessel at any given time.

The model used in this study is an extension of the model presented by Levin and Udekwu [42]. The model has been used to interpret the effects of periodic antibiotic treatment of *S. aureus* continuous cultures [39]. Briefly, the model assumes a 1 ml vessel into which resource *R* flows at a rate *w* from a reservoir with a resource concentration *C* of 500 (Fig. 4). Each bacterial population has unique growth and death rates described by Hill functions with parameters reflecting the susceptibility of the population to antibiotics [43]. The model also accounts for resource and density effects on antibiotic efficacy as well as density-dependent antibiotic decay.

In this extension of the model, there are four cell populations: *P*, planktonic bacteria; *Ag*, planktonic aggregators; *B*, bacteria within biofilms; and *D*, dead cells (Fig. 4). Cells can move from the planktonic state to the biofilm state at rates *yb* (*P*→*B*) or *yp* (*Ag*→*B*). Shedding from biofilms is also represented as a conversion rate between cell populations, *yw* (*B*→*P*) or *yd* (*B*→*Ag*). Aggregators differ from planktonic cells only in their ability to form biofilm, which is reflected in an increased rate of switching (*yp*>*yb*). Similarly, aggregators differ from cells within biofilms only in their susceptibility to flow out of the system, *w*. There is no switching between planktonic and aggregator populations; the aggregators are assumed to be a low frequency, second population.

Additionally, a phage population *Q* was added to the model. Phage can replicate within all three viable cell populations, though not with the same infection parameters. There is a unique adsorption rate γ for each cell population. It is assumed that *γ_Ag_*>>*γ_B_*>>*γ_P_*, which reflects the locally high cell densities of the aggregator and biofilm populations, the expected increased rate of contact in the aggregators due to movement within the system, and the experimentally determined adsorption rates for planktonic cells and aggregators. Phage can also bind to dead cells *D* at the same rate as planktonic cells. Each population, with the exception of *D*, also has a unique burst size, β. Latent period is not included in the model.

With these assumptions and definitions and those given in Fig. 4, the rates of change in the density of the bacteria and phage, and the concentration of resource and antibiotic are defined by the following equations.
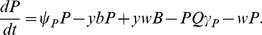
(2)

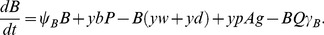
(3)

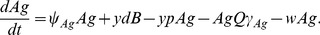
(4)

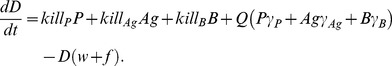
(5)

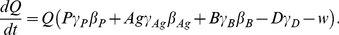
(6)

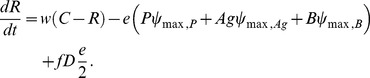
(7)

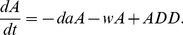
(8)where *ψ* is the realized growth rate of the given population, *kill* is the death rate of a given population, *e* is the conversion efficiency of the resource, *f* is the rate at which dead cells are converted into resource, and *ADD* is an operator that adds 100 xMIC of antibiotic every 24 hours. For all of the simulations reported here, initial *R* = 1, *M_min_* = 1, *M_max_* = 40, *pd* = 0, *cr* = 1, κ = 1, *e* = 5e7, *k_R_* = 0.25, *k_max_* = 1e7, *f* = 0, *ddx* = 0.01, *dv* = 0, *dk* = 1e-9, and *w* = 0.3 (see [42] for additional parameter definitions).

**Figure 5:**
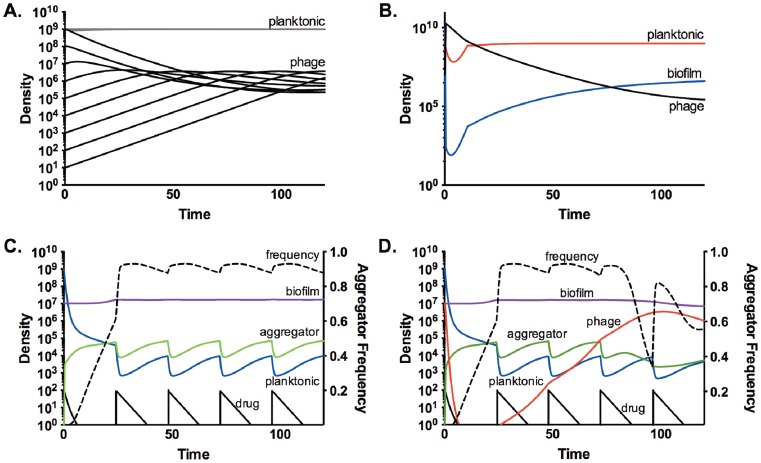
Computer simulations of single- and dual-treatment regimes. A. and B.) Model predictions of the population dynamics with phage treatment. Initial conditions: N = 1e9, R = 1, ψ_max_ = 1, ψ_max,B_ = 0.001, γ = 1e-10, γ_B_ = 1e-8, β = 2, β_B_ = 2, yb = 1e-4, yw = 1e-2. A.) Changes in phage (black) and planktonic cell (gray) densities with varying initial densities of phage, from 1e1 to 1e9. B.) Changes in planktonic (red), biofilm (blue) and phage (black) densities with a very high initial biofilm density (10^10^). C. and D.) Changes in the densities of planktonic (blue), biofilm (purple), and aggregator (green) cells. The black line is the drug concentration in xMIC. The frequency of aggregators in the planktonic compartment (i.e.-Ag/P+Ag) is shown (dotted line). Initial conditions: *P* = 1e9, *B* = 1e7, *Ag* = 1, *R* = 1, *A* = 100, *ψ_Max_* = 0.2, *ψ_Min_* = *ψ_Min, Ag_* = −3, *ψ_Max,Ag_* = *ψ_Max, B_* = 0.1, *ψ_Min, B_* = −0.001, γ = 1e-10, γ*_B_* = 1e-8, γ*_Ag_* = 1e-6, γ*_D_* = 6e-9, β = β*_B_* = 3, *yp* = 0.01, *yd* = *yb* = 0.001, *yw* = 0.0001. C.) Model predictions of the population dynamics in a GEN-treated continuous culture. D.) Model predictions of the population dynamics in a dual-treated continuous culture. The initial phage density (*Q*) was 1e7.

Using replication parameters which are consistent with a “typical” lytic phage (adsorption rate = 1×10^−9^ ml^−1^ hr^−1^, burst size = 100 phage), the simulation results in rapid clearance of the host cell population, followed by a slower loss of phage due to flow out of the system (data not shown). However, the experimental results in Fig. 1B can be recapitulated by this strictly lytic model when the burst size of the phage is very low (2 phage), the adsorption rate to planktonic cells is low (1×10^−10^ ml^−1^ hr^−1^) and the adsorption rate to biofilm cells is high (1×10^−8^ ml^−1^ hr^−1^) (Fig. 5A). In these conditions, the phage population stabilizes at ∼1×10^6^ phage/ml, regardless of the initial phage inoculum. Additionally, due to the very low adsorption rate, the planktonic population remains stable, with only very high phage densities resulting in transient perturbations. The initial perturbation of the planktonic density can be simulated if the initial biofilm density is very high (10^10^), but the system rapidly returns to its equilibrium state (*P* ∼ 1×10^9^, *B* ∼ 1×10^7^, and *Q* ∼ 1×10^6^) (Fig. 5B).

Simulations with drug treatment alone suggested that increased capacity to form biofilm can explain the rise of the aggregator population (Fig. 5C). By 48 hrs, the aggregator population, distinguished only by a higher rate of switching to the biofilm compartment, had ascended to dominance in the planktonic compartment, and this dominance was maintained. In the absence of drug treatment, the aggregator population did not ascend (data not shown), consistent with the absence of detectable aggregator isolates in the control and phage-only cultures. These simulations are consistent with the hypothesis that increased biofilm-forming capability alone can confer a fitness advantage in the presence, but not in the absence, of antibiotics.

The results of the simulation of dual-treatment are not as clear. The numerical model can approximate the experimental results of dual treatment only when the phage adsorption rate to aggregators is substantially higher than the adsorption rate to any other cell population (e.g.- *γ_Ag_*>>*γ_B_*>>*γ_P_*) and the phage can bind non-productively to dead cells (*D*) (Fig. 5D). As seen in the experimental system (Fig. 1C), the phage density declines rapidly, followed by a very slow recovery. However, the conditions for this result are very narrow. Small changes to the parameters result in one of two outcomes: 1) the low density in the planktonic compartment is maintained but the phage is lost, or 2) the density in the planktonic compartment recovers to ∼10^9^ and the phage is maintained (data not shown).

## Discussion

Understanding the pharmacodynamics of antibacterial treatment is critical to designing effective therapeutic regimens. This knowledge is even more important as we consider integrating infectious, replicating entities such as bacteriophage into treatment protocols. In this study, a continuous culture model system is presented along with a numerical computer model, which can be used in combination to examine the pharmacodynamics of a variety of potential therapies. Here, the system is used to evaluate the potential for lytic bacteriophage to augment antibiotic treatment.

As seen previously [39], daily treatment with gentamicin resulted in stable oscillations in the planktonic cell density (Fig. 1B). Unexpectedly, this treatment also induced the rise of a subpopulation of aggregator cells, which have increased capacity to form biofilms (Fig. 2). Recently, Haaber et al. reported the induction of aggregation in *S. aureus* as a result of sub-MIC exposure to several common antibiotics [44], suggesting that this physiological response is not unique to the aminoglycosides. In *S. aureus*, aggregation, as well as biofilm formation, has been linked to increased expression of surface carbohydrates [44] and surface proteins [45]. Preliminary data suggests that, in PS80, induction of aggregation is controlled by IcaR, and the phenotype is mediated by one or more surface proteins (data not shown). If one of these surface proteins is also the receptor for SA5, this would also explain the increased absorption rate to aggregators. It is tempting to speculate on the potential role of teichoic acids in this phenomenon, due to their role in biofilm formation [46], [47], [48] and aminoglycoside uptake [49]. However, this is unlikely, as the strictly lytic Myoviruses are not dependent on teichoic acids [50], [51]. Further research is needed to fully characterize the molecular mechanism of aggregator induction.

Aggregators did not contribute to population-level resistance to drug treatment via a measurable increase in MIC (this study) or survival when exposed to antibiotics during exponential phase [44]. Instead, as supported by the simulations (Fig. 5C), the increased biofilm capacity of aggregators is sufficient to allow their ascension in the culture. This result is consistent with the model findings in Udekwu and Levin that the biofilm population was necessary to support the cell density oscillations induced by several classes of antibiotics, ostensibly by reseeding the planktonic population [39].

The rise of the aggregator population is integral to the dynamics of the dual-treated cultures. In the first 24 hours, gentamicin reduces the cell density by approximately four logs (Fig. 1A, C). During this time, the phage density drops precipitously, most likely due to a combination of attachment to gentamicin-killed cells and flow out of the system. As the aggregators ascend in the culture, the phage density slowly begins to recover. The results of the dual-treatment model suggest that this temporal correlation in phage and aggregator ascendance is critical. In the model, the resurrection of the phage population is dependent upon the rise of aggregators, and is exquisitely sensitive to the infection parameters for these cells. Also, in replicate continuous cultures, when aggregators were not detected until very late (i.e.- 72 or 96 hrs after initiation), the phage population did not recover (data not shown).

Once the phage recovers to its equilibrium density (∼10^6^ pfu/ml), the increased efficiency of phage replication in the aggregator cells causes the aggregator population to drop below detection. This drop is temporally correlated with less recovery of planktonic density after each dose of antibiotic, seen as a dampening of the amplitude of the oscillations (Fig. 1C, 48–100 hrs). This would be expected from the predictions of Udekwu and Levin [39]; any reduction in the biofilm population would be reflected in the planktonic compartment as lower recovery after antibiotic dosing.

Although the dynamics of the dual-treated cultures could be reproduced in the model, the model predictions were highly sensitive to changes in parameter values. This instability could be due to the lack of structural parameters in the model. The structure of biofilms is only represented in the model by making the biofilm population independent of flow through the system. Since aggregators are modeled as a planktonic population, they are subject to flow and, thus, have no structural information in the model. However, in the experimental system, the aggregators, by definition, are in a more structured microenvironment than are the non-aggregator planktonic cells. Like attached biofilms, these aggregates have very high local cell density, which likely accounts for the increased efficiency of phage replication in these strains (Fig. 3A). It is this increased efficiency of replication that allows the aggregators to serve as a reservoir for the phage during gentamicin treatment.

The biphasic shape of the SA5 adsorption curve is unexpected, but these kinetics have been reported for other bacteriophage [52], [53], [54], [55]. The two phases of adsorption have been hypothesized to be due to heterogeneity in the virus population, heterogeneity of receptors in the cell population, and/or fast, reversible attachment facilitating slower, irreversible attachment. Although none of these possibilities can be eliminated, the switching of the fast and slow phases in the aggregator strains suggests that, for SA5, the biphasic adsorption kinetics are largely due to heterogeneity of the cellular receptors for the phage. This is consistent with the changes in expression levels of multiple cell surface proteins in aggregators relative to PS80 (data not shown).

With antibiotic resistance increasing, there has been a renewed interest in adapting bacteriophage for use as antibacterials. Indeed, ISP, the closest relative of SA5, is being developed as a potential treatment for burn wound infections [41], [56]. One of the major limitations of phage-based therapies is the ability of the bacteria to become resistant to the phage. Even with an arms race scenario, where phage-resistance mutations are countered with host-range phage mutants, ultimately the phage cannot continue to change and resistant bacteria dominate the population [20], [21], [22], [23], [57]. Combined therapy with phage and antibiotics has been suggested as a way to control the impact of resistance, to both phage and antibiotics. Ideally, the combination would be designed such that resistance to one agent increases the sensitivity to the other. The results of this study support the feasibility of this “double bind” approach. In this case, the cellular changes necessary to survive the antibiotic treatment predisposed the cells to phage predation, and ultimately, the combined treatment resulted in more bacterial killing with no detectable phage or antibiotic resistance.

The synergistic action of antibiotic dosing and bacteriophage treatment described here is likely dependent on the specific combination of antibiotic, bacteriophage, and bacterial strain. However, evidence suggests that the underlying microbiological phenomena are not unique to the components chosen for this study. The aggregation phenotype has been reported in many *S. aureus* strains, including clinical isolates [44], [45], [58], [59]. Similar aggregation phenotypes have been reported in *Escherichia coli* [60], *Campylobacter jejuni* [61], *Streptococcus pyogenes* [62], and *Pseudomonas aeruginosa* [63]. This phenotype is not likely an artifact of laboratory culture, as it has been found in active *P. aeruginosa* infections in the lungs of cystic fibrosis patients [64]. Although some bacterial species and strains aggregate without induction, a variety of antibiotics and antimicrobials have been reported to induce aggregation [44], [59], [60]. In addition, the closest relative of SA5, bacteriophage ISP, has a broad host range. It was able to infect 74 out of 86 *S. aureus* strains tested, including all of the human isolates [41]. With a very high degree of identity and differences restricted to single point mutations distributed across the genome, SA5 is expected to have a similarly broad host range. Thus, the synergy described here, while not expected to be universal, is likely to be common enough to warrant further study as a potential treatment modality. This study presents both a proof-of-principle for the double bind approach to phage-antibiotic dual therapy and a model system with which to test such protocols.
